# Kind Words and Suggestions from Friends

**Published:** 1872-11

**Authors:** 


### KIND WORDS AND SUGGESTIONS FROM FRIENDS.

We publish the following extracts because we feel that these "kind words" have done us good, and we hope and believe they will do our readers good. We desire, earnestly, to make the COMPANION as much the journal of our readers as it is that of the Editors. We wish all of them to feel that it is *their journal;* that through it they can always speak. Kind words and suggestions are ever welcome from our friends:

"LET US HAVE PEACE."

"The glory and prosperity of the COMPANION lies in its former popular style of articles and the brevity, and great variety of them. Play to the tune of a simple English style, brevity and plenty of articles, and you will find the richest "California diggings."

"Wishing you health, strength and prosperity. God's blessings, yours, etc." * * *.

WE thank the distinguished writer for the above kind expressions and his wholesome advice. The COMPANION will devote itself to "brevity and plenty of articles" in the future, which we are glad to know has given it "glory and prosperity." The present number, we think, will bear testimony to our efforts in this direction.

---

"Your letter to the editor of the "Largest Medical Journal in America," published in the September number, is sufficient. Say no more. We are all satisfied, in our city, that no one connected with the COMPANION would have a franchise changed without the consent of the corporators, forge a letter or make a misstatement to get in or out of a trouble, not even to be made Emeritus Professor. Do your best to get up the most practical and useful journal in America, and you will have no further trouble." * *

WE assure our brother co-laborer we are "law-abiding" men. He and all friends may rely upon the truth of all statements made in the COMPANION. If any statement is not in strict harmony with fact, we will take the greatest pleasure in making the correction—as we desire simply to tell the truth and nothing more. We are glad we are so fully understood, and our object so generally appreciated. Having no disposition to discuss "taste" or any personal issues, we shall, in the future, seek to make the Companion all that its friends may desire.

---

"We are more and more pleased with the Companion. Pledge yourself to print it on better paper and mail it punctually, and you will double your list of subscribers by the time you can get out the January number. I am certain every subscriber can and will send you one new one if you will do as I have suggested. I will promise *five*. We must have one independent Southern Journal. If you can't send specimen numbers, send Circulars to our Northern and Western brethren. I know they will help us."

We can get up the Companion in the very best style—with the best material and paper—if our friends will second the efforts of our Virginia correspondent. Let every gentleman who is now a subscriber, and who feels an interest in the Companion, and desires to have it improved, adopt the suggestions above and send in the names of all new subscribers at once. This is necessary, as we wish to know the number of copies to be struck; and it is important every subscriber should commence with the beginning of the volume. Go to work, friends, and procure at least one new subscriber, and as many more as possible, and we will pledge ourselves to make our best efforts to furnish a journal punctually every month worth more than the subscription price, and at the end of the year the most practical, useful and valuable volume ever published in this country.

